# Transcatheter closure of a quadriform atrial septal defect with a custom double-fenestrated occluder: A case report

**DOI:** 10.20407/fmj.2026-005

**Published:** 2026-09-25

**Authors:** I Ketut Alit Utamayasa, Alisia Yuana Putri, Dian Paramita Kartikasari, Zakirah Zuhra

**Affiliations:** 1 Faculty of Medicine, Airlangga University, Surabaya, East Java, Indonesia; 2 Department of Pediatrics, Dr. Soetomo General Hospital, Surabaya, East Java, Indonesia; 3 Department of Cardiology and Vascular Medicine, Dr. Soetomo General Hospital, Surabaya, East Java, Indonesia

**Keywords:** Atrial septum, Catheterization, Pulmonary arterial hypertension

## Abstract

**Background:**

Quadriform atrial septal defect (ASD) is a rare anatomical and functional variant of ASD. Its rarity and complex morphology pose significant diagnostic and therapeutic challenges, distinguishing it from more common forms of ASD. Although surgical closure has traditionally been the standard treatment, this case demonstrates the feasibility and efficacy of a minimally invasive transcatheter approach.

**Case Report:**

A 71-year-old woman presented with shortness of breath. Physical examination and transthoracic echocardiography revealed an interatrial septal defect. Right heart catheterization combined with transesophageal echocardiography confirmed a complex ostium secundum ASD with a cribriform morphology resembling a quadriform ASD and associated high-flow, low-resistance pulmonary hypertension. Device deployment required additional manual modification and manipulation by the operators. Successful ASD closure was achieved using a single 40-mm occluder with double fenestrations. The patient remained asymptomatic at the 3-month follow-up.

**Conclusion:**

Transcatheter device closure may be an effective treatment option for quadriform ASD, although close follow-up is required to ensure proper device positioning. The increasing complexity of ASD presentations, particularly secundum defects, underscores the importance of continued discussion and experience sharing within the cardiovascular community.

## Background

Atrial septal defects (ASDs) represent a common form of congenital heart disease, characterized by defects in the interatrial septum that allow communication between the right and left atria.^1^ Recent advancements in minimally invasive transcatheter procedures, specifically the use of occluder devices, have enhanced the approach to ASD closure, offering promising results in terms of efficacy and safety.^2^ Transcatheter ASD closure is favored because of its reduced hospitalization time and morbidity compared with surgery.^3^

In the present case, the unusual defect morphology required the operators to perform additional maneuvers during device deployment. Centrally located secundum defects are ideal for device closure, but there is considerable morphological variation in the size and location of these defects.^4^ A cribriform ASD resembling a quadriform ASD is an unusual ASD morphology. Because of the rarity of this condition, the management of quadriform ASD remains individualized and patient-specific. Older adults may initially remain asymptomatic but frequently develop significant morbidities, including exercise intolerance and heart failure, with advancing age. Transcatheter closure may be an alternative to open heart surgery in these patients.^2^

## Case Report

A 71-year-old Asian woman presented with shortness of breath. The patient had experienced shortness of breath for approximately 10 years, which had worsened during the 5 months prior to admission. She had no comorbidities, past medical history, or previous interventions. Physical examination revealed a grade IV/VI holosystolic murmur heard loudest at the fourth intercostal space along the left parasternal line. Oxygen saturation was 96% on room air. Chest X-ray showed the trachea in the midline, no bony abnormalities, cardiomegaly (cardiothoracic ratio of 87%), widening of the right cardiac border laterally, a prominent conus pulmonalis, and an obtuse right costophrenic angle, while the left costophrenic angle was obscured by the cardiac silhouette (Figure 1). Transthoracic echocardiography revealed an interatrial septal defect, moderate probability of pulmonary hypertension, reduced left ventricular dimensions, and dilation of the pulmonary artery, right atrium, right ventricle, and left atrium.

Given the chronic nature of her symptoms, right heart catheterization (RHC) was performed. It demonstrated multiple ASDs with high-flow, low-resistance pulmonary hypertension. The patient underwent transesophageal echocardiography (TEE) during RHC, which revealed a quadriform ASD with a left-to-right shunt. The defect measured 17–27 mm by two-dimensional imaging and 22×29 mm by three-dimensional imaging. Rim measurements were as follows: atrioventricular rim, 22 mm; posterior rim, 16 mm; aortic rim, 5 mm; IVC rim, 35 mm; and SVC rim, 18 mm. Severe tricuspid regurgitation (TR), a high probability of pulmonary hypertension, and aortic valve calcification without stenosis or regurgitation were also observed (Figure 2).

TEE performed during RHC demonstrated a complex ostium secundum ASD with four distinct fenestrations within the atrial septum, consistent with a cribriform morphology. In this report, we use the term “quadriform ASD” to describe this complex ostium secundum variant. The overall ASD diameter was 20×26–29 mm by three-dimensional imaging. The posterior rim measured 16 mm, aortic rim 5 mm, SVC rim 18 mm, IVC rim 20–35 mm, and atrioventricular rim 22 mm. Additional findings included severe TR, right atrial and right ventricular dilation, left atrial appendage spontaneous echo contrast, and trivial mitral regurgitation. RHC demonstrated a right atrial pressure of 21 (a-wave)/ 26 (v-wave) (22) mmHg, left atrial pressure of 20 (a-wave)/ 28 (v-wave) (22) mmHg, pulmonary artery pressure of 106/24 (59) mmHg, aortic pressure of 96/43 (61) mmHg, flow ratio of 1.449, pulmonary vascular resistance (PVR) of 4.64 WU, PVR index of 3.03 WU·m^2^, and PVR/systemic vascular resistance (SVR) ratio of 0.55. An oxygen test was performed to assess pulmonary vascular reversibility. The patient received 100% oxygen for 10 minutes via intubation. After oxygen administration, right atrial pressure was 21 (a-wave)/ 26 (v-wave) (22) mmHg, left atrial pressure was 22 (a-wave) /30 (v-wave) (24) mmHg, pulmonary artery pressure was 103/26 (56) mmHg, aortic pressure was 105/52 (72) mmHg, flow ratio was 3.312, PVR was 0.73 WU, PVR index was 1.12 WU·m^2^, and the PVR/SVR ratio was 0.138. These findings were consistent with high-flow, low-resistance pulmonary hypertension (Table 1). The patient was considered a suitable candidate for transcatheter device closure because of the ASD anatomy and the presence of high-flow, low-resistance pulmonary hypertension.

By considering the patient’s age, the overall diameter of the ASD, sufficient rim length, and low-resistance pulmonary hypertension, the operators decided to perform transcatheter ASD closure using a single occluder device. Successful closure was achieved using a 40-mm standard ASD occluder with double fenestrations under general anesthesia with angiographic and TEE guidance (Figure 3).

The fenestrated occluder was manually prepared by the operators before implantation. Given the unavailability of a fenestrated Amplatzer Septal Occluder (ASO) in our region and because of device limitations, the ASO was manually perforated to create the fenestrations. The ASO was punctured using an 18-gauge needle. A guidewire was advanced, followed by insertion of a dilator from a 6F sheath. The 6F sheath dilator and guidewire were then withdrawn. A hydrophilic wire was subsequently introduced, followed by advancement of a dilator and a 14F sheath. All instruments were then removed. The procedure was repeated twice, resulting in a double-fenestrated ASO.

Post-closure TEE demonstrated that the ASD occluder device was well positioned, with no residual shunt. Although fenestrations were intentionally created in the occluder device, no significant residual shunt was detected on immediate postprocedural imaging. This finding may be explained by temporary functional closure or imaging limitations. No arrhythmias or conduction abnormalities were reported. Closure of the ASD resulted in significant clinical improvement. The patient was prescribed an aldosterone antagonist, angiotensin-converting enzyme inhibitor, antiplatelet agent, and phosphodiesterase type 5 inhibitor for daily use.

Severe TR before the procedure was attributed to annular dilation, with a maximum TR pressure gradient of 113 mmHg. After device closure, severe TR persisted, with a maximum TR pressure gradient of 102 mmHg on transthoracic echocardiography. At 1 month after closure, severe TR remained, with a maximum TR pressure gradient of 119 mmHg. The patient received optimal medical therapy and close monitoring to prevent right heart failure secondary to severe TR, including treatment with a diuretic and an angiotensin-converting enzyme inhibitor. The patient remained asymptomatic, and no complications were observed during the 3-month follow-up.

## Discussion

Quadriform ASDs are a type of multi-fenestrated ASD, and their anatomical and functional characteristics are important considerations in treatment planning.^2,4^ The spatial relationship and distance between adjacent defects are key morphological features. If the interdefect distance exceeds 7 mm, coverage of all defects with a single closure device is not recommended.^4^

Acute hemodynamic decompensation is a significant concern.^5^ In older adults with poor left ventricular compliance, ASD closure may increase the risk of acute pulmonary edema and heart failure. Moreover, long-term shunting in older patients often leads to irreversible structural heart damage.^2,5^ A systematic review indicated that even after closure, complications such as persistent right ventricular dilation and TR may persist.^2^ Nevertheless, mid- and long-term survival after ASD correction in adults is now considered comparable to that of the general population, regardless of age.^2^

Transcatheter closure of multiple or fenestrated ASDs is an established alternative to surgical repair, although it presents unique technical challenges.^1^ The literature includes reports of multiple defects being successfully closed using multiple devices. For instance, a case report by Cho et al. described two cases of multiple ASDs closed with two separate Amplatzer devices.^1^ In one case, two defects 15 mm apart were closed with 24-mm and 8-mm devices, respectively. In the second case, which involved three defects, a 35-mm cribriform occluder was used to cover two closely spaced defects (separated by a 3.5-mm septum), while a third, more distant defect (7 mm away) was closed with a separate 12-mm device.

Studies indicate that transcatheter device closure can achieve satisfactory outcomes with low complication rates when performed by experienced operators.^3,4^ As highlighted in the literature, complications related to occluder device embolization and malposition necessitate careful patient selection and meticulous procedural technique. Despite the advantages of device closure, inherent risks of postoperative complications remain and may affect long-term patient prognosis.^6^ Research suggests that persistent arrhythmias after device closure may result from atrial remodeling associated with the defect itself, independent of the closure procedure.^2^ Accordingly, understanding the interplay between ASD morphology, device characteristics, and patient-specific factors remains essential for the optimal management of these congenital anomalies.^1,7^

Transcatheter closure of ASDs primarily utilizes devices such as the ASO, which is specifically designed to accommodate variability in defect morphology. Large ASDs, which often lack adequate rims, pose significant challenges for device deployment.^8^ Further work by Takaya et al. confirmed these findings by describing innovative techniques using steerable guide catheters to navigate complex venous anatomy during ASD closure, reinforcing the versatility required in such procedures.^9^ Midazolam has also been reported as an effective agent for conscious sedation during transcatheter closure guided by transthoracic echocardiography, demonstrating its role in enhancing patient comfort and procedural efficacy.^10^ Complications following ASD device closure remain a substantial focus of the current literature.^11,12^ Studies such as that by Tanghöj et al. have examined the risks of postoperative events, including arrhythmias.^11^ In contrast to conventional approaches, intraoperative closure performed under TEE guidance has been associated with favorable complication rates and outcomes.^13^ Additionally, comparative studies assessing transcatheter and transthoracic device closure have shown that although both methods are effective, transcatheter approaches often result in shorter recovery times and less invasive treatment experiences.^3,14^ Although percutaneous closure of complex ASDs has become an established treatment option, ongoing research is needed to address complications and further refine procedural techniques. As advancements continue, patient-centered care that incorporates individual anatomical considerations and potential risks will remain paramount.^15^

## Conclusion

Quadriform ASD presents a challenging diagnostic and therapeutic challenge. Traditionally, management has involved surgical intervention. This case highlights the effectiveness of a minimally invasive transcatheter device closure approach, avoiding extensive surgical trauma, even in a patient of advanced age with an unusual ASD morphology. However, ASD management remains complex and requires consideration of risks such as peridevice leakage and device malposition after closure. Close follow-up with physical examination and echocardiography is essential to ensure proper device positioning and sustained symptom relief. Further research is warranted to clarify the optimal treatment strategies and long-term outcomes of single-device transcatheter closure in patients with unusual ASD morphologies.

## Figures and Tables

**Figure 1:**
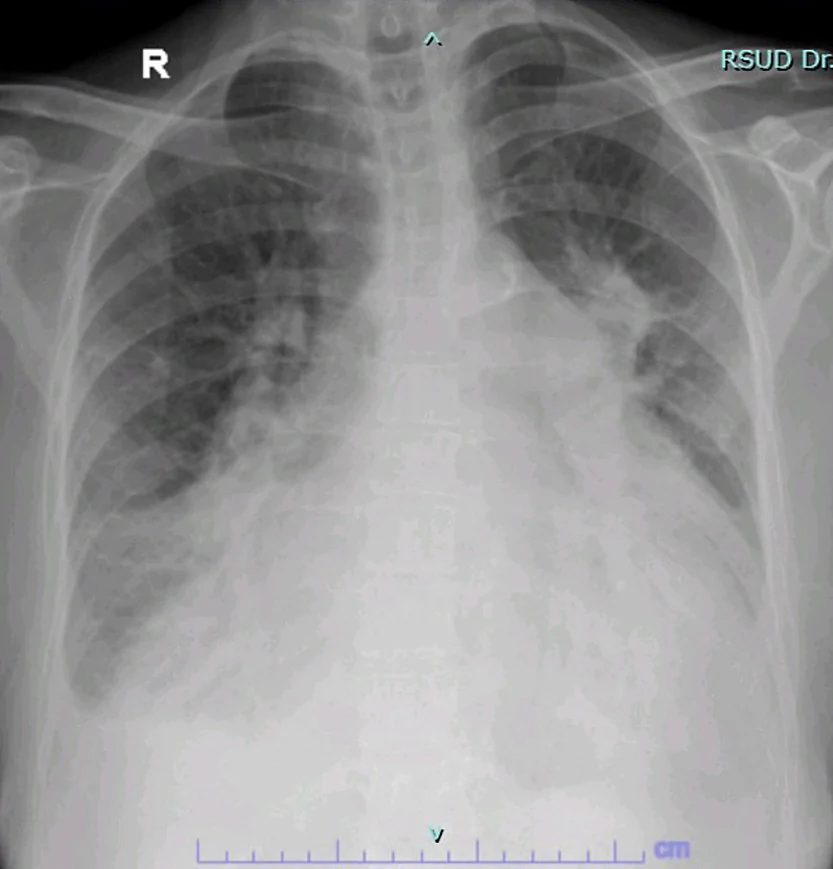
Chest X-ray showing cardiomegaly

**Figure 2:**
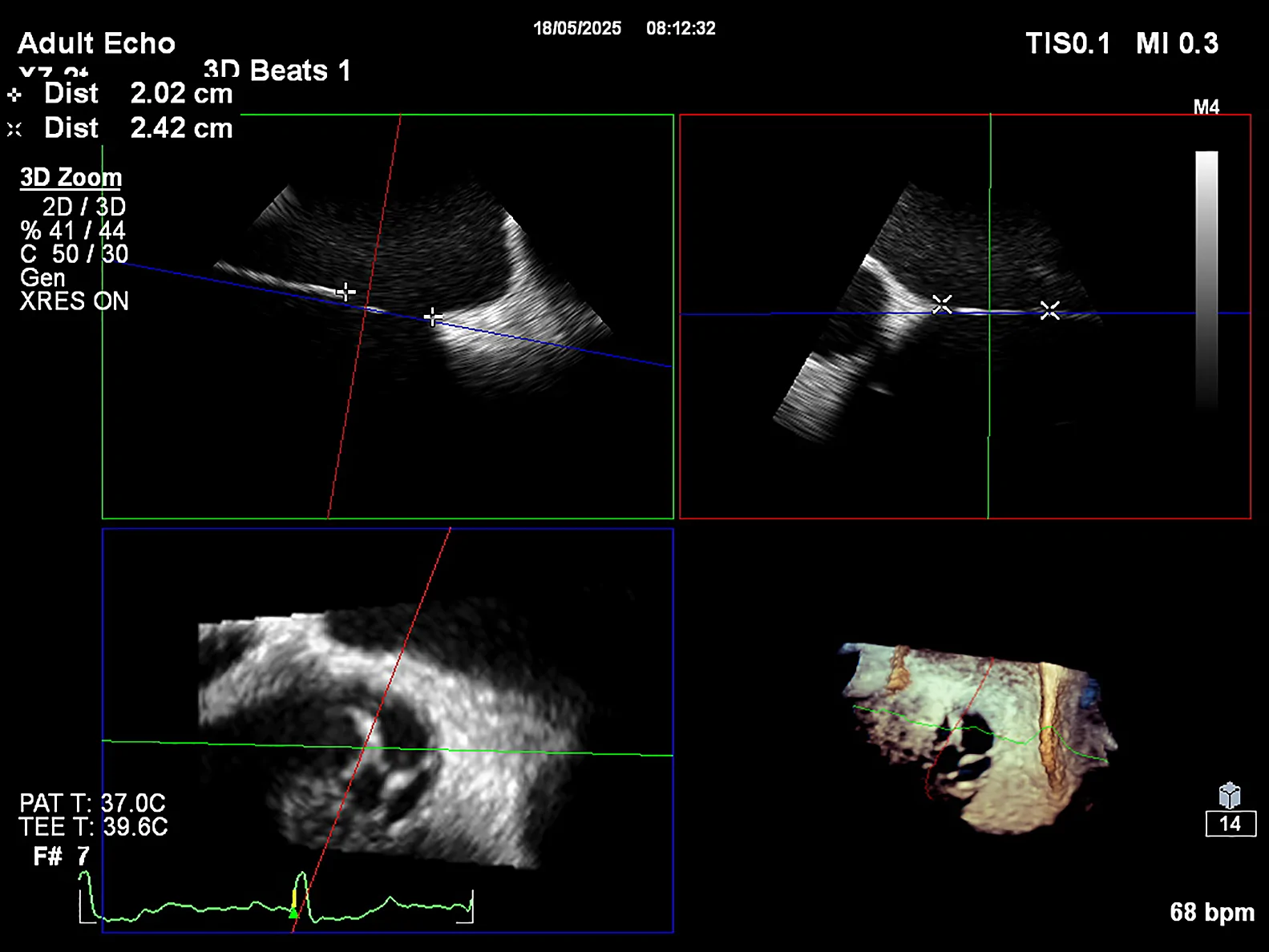
Transesophageal echocardiography showing multiple atrial septal defects

**Figure 3:**
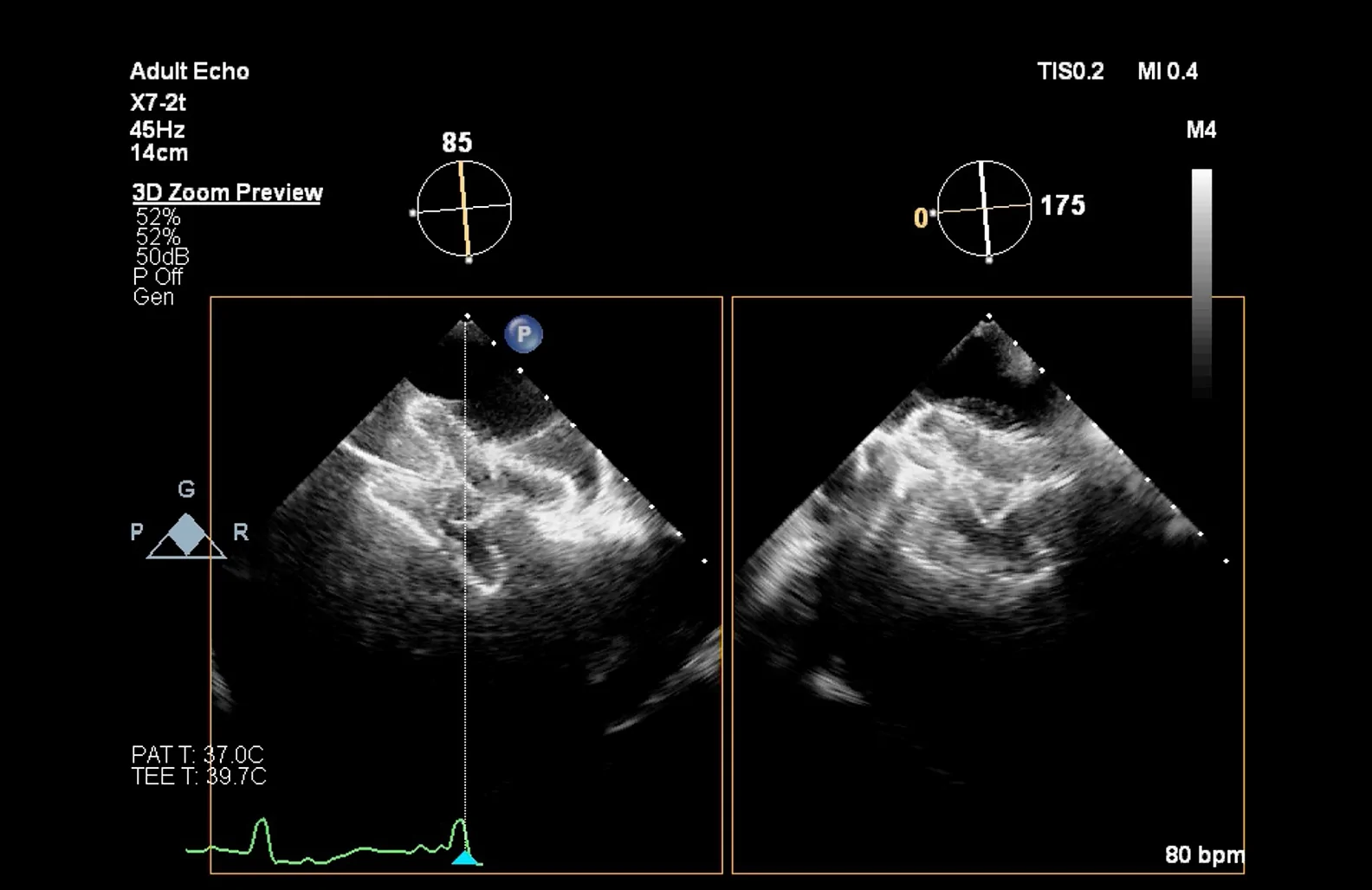
Transesophageal echocardiography showing the occluder device in place

**Table 1:** Cardiac catheterization results

Site	Pre-O_2_				Post-O_2_		
	Phasic (mmHg)	SpO_2_ (%)	Hb (g/dL)		Phasic (mmHg)	SpO_2_ (%)	Hb (g/dL)
High SVC		69.2	10.2			80.3	10.0
Low SVC		69.5	10.0			80.0	10.0
High IVC		73.0	9.4			82.3	9.8
RA	21/26 (22)	75.4	10.0			89.5	9.0
PA	106/24 (59)	76.5	10.5		103/26 (56)	89.9	9.9
Left upper PV		92.0	10.6			94.0	10.4
LA	20/28 (22)	92.0	10.1		22/30 (24)	93.4	10.3
Ao	96/43 (61)	92.0	8.5		105/52 (72)	94.2	9.6

Ao, aorta; Hb, hemoglobin; IVC, inferior vena cava; LA, left atrium; PA, pulmonary artery; PV, pulmonary vein; RA, right atrium; SpO_2_, oxygen saturation; SVC, superior vena cava
